# Successful monotherapy with autologous formalin‐fixed tumor vaccine for a Stage IV uterine cancer patient who rejected rational chemotherapy and immune checkpoint inhibitor treatment

**DOI:** 10.1002/ccr3.7513

**Published:** 2023-06-08

**Authors:** Katsuhiko Fukuda, Tadao Ohno

**Affiliations:** ^1^ Integrated Medical Center Fukuda Internal Medicine Clinic Matsue‐shi Japan; ^2^ Cell‐Medicine, Inc. Tsukuba Science City Japan

**Keywords:** cancer vaccine, immunotherapy, monotherapy, uterine cancer

## Abstract

**Key Clinical Message:**

To overcome patient‐initiated treatment refusal because of fear of experiencing severe negative adverse events, mild immunotherapy using a cancer vaccine such as the autologous formalin‐fixed tumor vaccine should be considered.

**Abstract:**

A patient who refused chemotherapy and immune checkpoint inhibitor treatment for Stage IV uterine cancer after displaying circulating tumor cells and high microsatellite instability received monotherapy with autologous formalin‐fixed tumor vaccine (AFTV). Following treatment, we observed regression of multiple lung metastases, suggesting that AFTV is an attractive treatment option.

## Graphical abstract

XXX

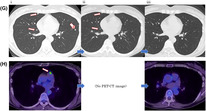

## 1 INTRODUCTION

In clinical settings for cancer patients, clinicians may have experienced a strong rejection of rational treatment because of the patient's fear of severe adverse effects (SAEs) of the proposed chemotherapy and/or immune checkpoint inhibitors. Autologous formalin‐fixed tumor vaccines (AFTV) are treatment options that have been exclusively trialed in Japan and Germany. Here, we report a patient with Stage IV uterine carcinoma who initially rejected chemotherapy and anti‐PD‐1 antibody treatment despite the presence of apparent circulating tumor cells (CTC) that were sensitive to paclitaxel and carboplatin and carried microsatellite instability (MSI).

## 2 CASE REPORT

**FIGURE 1:**
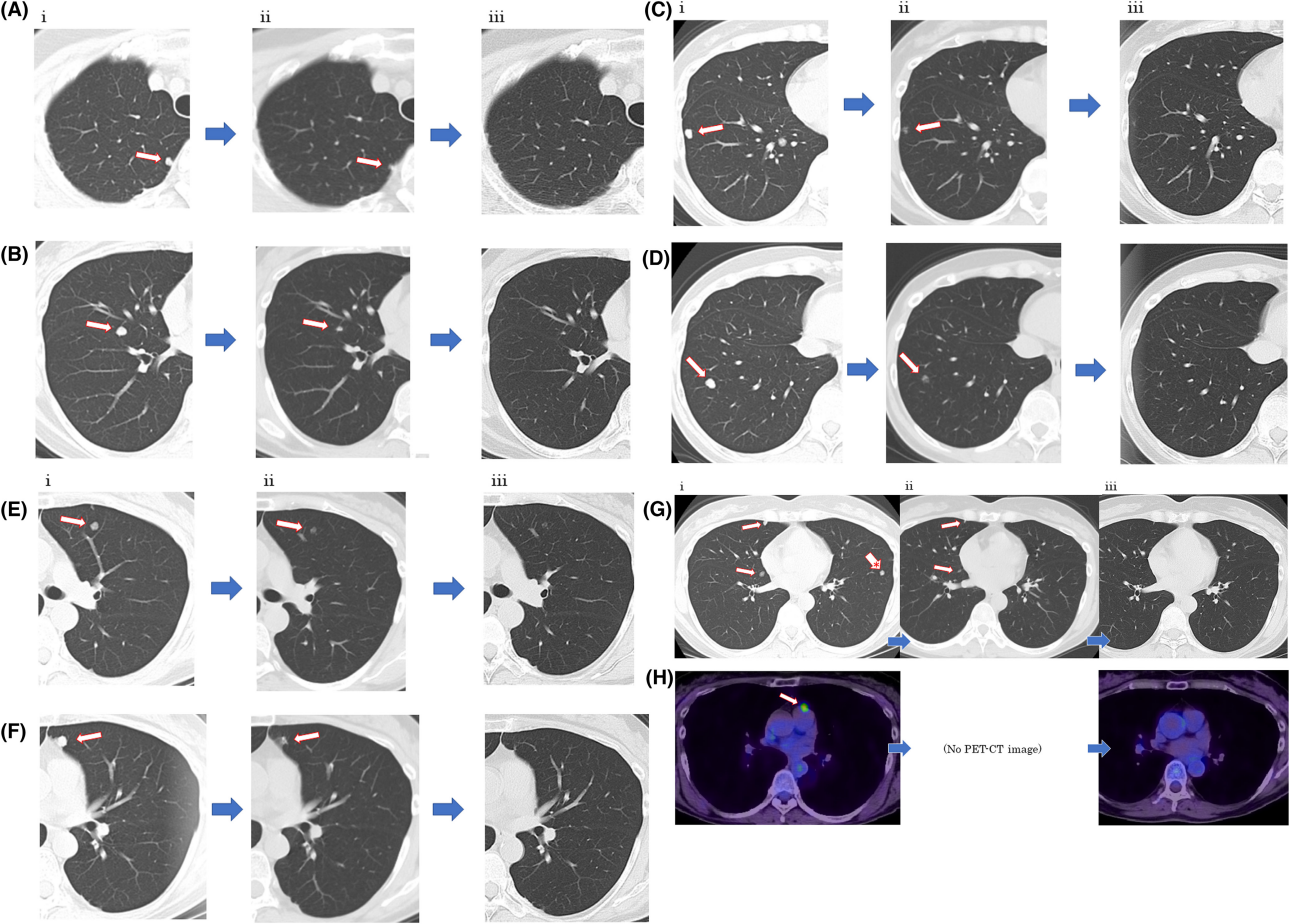
CT images of the lung and PET‐CT images of a para‐aortic lymph node. (A–G) Series i: CT images before AFTV treatment. Nine metastases (arrows) of uterine cancer are observed. (H, i) PET‐CT image indicating a small hotspot at the para‐aortic lymph node (arrow). (A–G) Series ii: Images after initial AFTV monotherapy. Three intradermal AFTV injections are administered 2‐week interval between each dose. The metastasis indicated with a red‐asterisk arrowhead (G, i) disappeared in (G, ii). No PET/CT images are available at H or in Series ii. (A–H) Series iii: Images after a one‐time injection of chemotherapy with carboplatin and docetaxel plus two additional injections with AFTV. A complete response is observed.

A 56‐year‐old female was diagnosed with uterine endometrioid adenocarcinoma G3, Stage IB (T1bN0M0) in April 2020. The patient underwent simple hysterectomy, bilateral salpingo‐oophorectomy, greater omentum segmental resection, and lymphadenectomy of the pelvis, paraaortic, and retroperitoneum. The patient adamantly refused standard chemotherapy and treatment with immune checkpoint inhibitors. Her follow‐up CTC level was high (6.2 cells/6.5 mL), as detected by Oncocount RGCC, Research Genetics Cancer Center (RGCC International GmBH, Switzerland). Pathology tests revealed her CTC was sensitive to chemotherapeutic agents, including carboplatin and docetaxel, and MSI‐high. Computed tomography (CT) revealed multiple metastases on both sides of her lungs (Figure 1A–G, Series i) and a positive signal in para‐aortic lymph nodes on the hilum of the left kidney on positron emission tomography (PET)‐CT (Figure 1H, series i) in October 2020. This indicated the tumors had metastasized to Stage IVB. Therefore, we selected AFTV treatment.

From November 2020 to January 2021, she received three intradermal injections of AFTV with a 2‐week interval between each dose. AFTV was prepared from the patient's own resected formalin‐fixed uterine carcinoma tissue, similar to the procedure reported for glioblastoma.^1^ No SAEs were observed and the adverse events reported included erythema and induration at the injection sites (all less than Common Terminology Criteria for Adverse Events, v4.0, grade 2). Subsequently, CT imaging revealed a reduction of nine lung metastases in December 2021, as shown in Figure 1A–G. In particular, the lung metastasis observed in Figure 1G, Series i (arrowhead with an asterisk) disappeared in Figure 1G, Series ii. This suggests that AFTV monotherapy is efficacious in treating lung metastases.

She reluctantly accepted a single dose of standard chemotherapy with carboplatin and docetaxel (70 mg/m^2^ and AUC 5, respectively) at a regional hospital to suppress the remaining metastases. This resulted in several typical SAEs such as nausea, diarrhea, stomatitis, anorexia, numbness, psilosis, and transient leukopenia. She quickly rejected further chemotherapy and was then switched back to two more AFTV injections in January and February of 2021 in our clinic. CT and PET‐CT images revealed a complete response of the lung and para‐aortic lymph node metastases to AFTV, as shown in Figure 1A–H, Series iii. As of June 11, 2022, she was clinically stable.

## 3 DISCUSSION

To the best of our knowledge, with the exception of AFTV injections, monotherapy of Stage IV carcinoma with cancer vaccines has yielded poor results. AFTV made from resected formalin‐fixed and paraffin‐embedded autologous tumor tissue has been effective in glioblastoma,^1^, ^2^, ^3^, ^4^ bone‐metastatic triple‐negative breast cancer,^5^, ^6^ upper tract urothelial carcinoma,^7^ advanced hepatocyte carcinoma,^8^, ^9^ malignant histiocytoma,^10^ peritoneal serous carcinomas recurrent after chemotherapy,^11^ gallbladder cancer,^12^ advanced colon cancer,^12^ and uterine cervical small cell carcinoma.^13^ Almost all of these tumors at advanced stages are known to be refractory to standard chemotherapy and immune‐checkpoint inhibitors. In addition, a randomized Phase II clinical study revealed statistical differences in recurrence‐free survival and overall survival in postsurgical hepatocellular carcinoma following treatment with AFTV.^14^

In the present case of advanced uterine carcinoma, early refusal of standard chemotherapy (carboplatin‐docetaxel, only one injection) may have preserved healthy functionality of her bone marrow, from which immune‐competent T lymphocytes were released without impairing cell proliferation and differentiation capacity. AFTV is capable of stimulating these cells to differentiate into cytotoxic T lymphocytes (CTL) in vivo.^9^ The basic mechanism of CTL induction of formalin‐fixed paraffin‐embedded tumor tissue sections has been shown in our previous publication.^15^

Although monotherapy with AFTV comprised three injections in the present case, the treatment resulted in 100% of the lung metastases reducing to less than half of their original size (Figure 1, Series ii). Although we are unable to conclude that the complete response of the lung metastases (Figure 1, Series iii) and the para‐aortic lymph node (Figure 1h, Series iii) are solely because of monotherapy with AFTV, the course of the present case strongly implies that AFTV monotherapy combined with one injection of cytotoxic agents (if required) will be an attractive treatment option for patients with severe adverse effects of standard chemotherapy. An official randomized clinical trial with appropriate control patients is required to confirm the efficacy of AFTV.

If AFTV monotherapy also becomes ineffective, we should consider that the recurrent/metastatic carcinoma containing mutant cancer cells have evaded immune control by AFTV‐induced CTL cells. Therefore, we need to prepare a new AFTV based on immuno‐escaped carcinoma, which may express novel neoantigens.

## 4 CONCLUSION

Treatment with AFTV is suitable for patients who reject standard chemotherapy for advanced cancer.

## AUTHOR CONTRIBUTIONS

**Katsuhiko Fukuda:** Conceptualization; data curation; investigation; resources; visualization; writing – review and editing. **Tadao Ohno:** Formal analysis; methodology; project administration; supervision; writing – original draft; writing – review and editing.

## FUNDING INFORMATION

No source of funding was declared for this study.

## CONFLICT OF INTEREST STATEMENT

The authors have no conflict of interest to declare.

## ETHICS STATEMENT

The ethics committee of Cell‐Medicine, Inc. has allowed us to co‐work with Integrated Medical Center Fukuda Internal Medicine Clinic on the autologous formalin‐fixed tumor vaccine (AFTV) treatment.

## CONSENT

Written informed consent was obtained from the patient for publication of this report according to the journal's patient consent policy.

## CLINICAL TRIAL REGISTRATION

The treatment described above has been carried out in daily clinical practice.

## Data Availability

The data that support the findings of this study are available from the corresponding author upon reasonable request.
